# Network-based quantitative trait linkage analysis of microbiome composition in inflammatory bowel disease families

**DOI:** 10.3389/fgene.2023.1048312

**Published:** 2023-01-23

**Authors:** Arunabh Sharma, Olaf Junge, Silke Szymczak, Malte Christoph Rühlemann, Janna Enderle, Stefan Schreiber, Matthias Laudes, Andre Franke, Wolfgang Lieb, Michael Krawczak, Astrid Dempfle

**Affiliations:** ^1^ Institute of Medical Informatics and Statistics, Kiel University, Kiel, Germany; ^2^ Institute of Medical Biometry and Statistics, University of Lübeck, Lübeck, Germany; ^3^ Institute of Clinical Molecular Biology, Kiel University, Kiel, Germany; ^4^ Institute for Medical Microbiology and Hospital Epidemiology, Hannover Medical School, Hannover, Germany; ^5^ Institute of Epidemiology, Kiel University, Kiel, Germany; ^6^ Department of Internal Medicine I, University Hospital Schleswig-Holstein, Kiel, Germany; ^7^ Institute of Diabetology and Clinical Metabolic Research, Kiel University, Kiel, Germany

**Keywords:** linkage analysis, gut microbiome, inflammatory bowel disease, family-based study design, microbiome co-occurrence network

## Abstract

**Introduction:** Inflammatory bowel disease (IBD) is characterized by a dysbiosis of the gut microbiome that results from the interaction of the constituting taxa with one another, and with the host. At the same time, host genetic variation is associated with both IBD risk and microbiome composition.

**Methods:** In the present study, we defined quantitative traits (QTs) from modules identified in microbial co-occurrence networks to measure the inter-individual consistency of microbial abundance and subjected these QTs to a genome-wide quantitative trait locus (QTL) linkage analysis.

**Results:** Four microbial network modules were consistently identified in two cohorts of healthy individuals, but three of the corresponding QTs differed significantly between IBD patients and unaffected individuals. The QTL linkage analysis was performed in a sub-sample of the Kiel IBD family cohort (IBD-KC), an ongoing study of 256 German families comprising 455 IBD patients and 575 first- and second-degree, non-affected relatives. The analysis revealed five chromosomal regions linked to one of three microbial module QTs, namely on chromosomes 3 (spanning 10.79 cM) and 11 (6.69 cM) for the first module, chr9 (0.13 cM) and chr16 (1.20 cM) for the second module, and chr13 (19.98 cM) for the third module. None of these loci have been implicated in a microbial phenotype before.

**Discussion:** Our study illustrates the benefit of combining network and family-based linkage analysis to identify novel genetic drivers of microbiome composition in a specific disease context.

## 1 Introduction

Inflammatory bowel disease (IBD) is a chronic inflammatory condition of the gastrointestinal tract that currently affects 6.8 million people worldwide (Alatab et al., 2020). IBD has two main sub-entities, Crohn disease (CD) and ulcerative colitis (UC), both of which are characterized by a complex etiology involving host genetics, microbiome and environmental factors (Lavoie et al., 2019; Caruso et al., 2020). Genome-wide association studies (GWAS) identified over 200 genetic variants that increase IBD risk (Lloyd-Price et al., 2019). In addition, IBD is also associated with a reduction of gut microbial diversity in the form of a decreased abundance of beneficial bacteria, such as *Faecalibacterium prausnitzii* and *Roseburia intestinalis*, and an increased abundance of harmful species (Ott et al., 2004; Imhann et al., 2018).

Several links have been drawn between host genetic variation and gut microbiome composition in the context of IBD (Bonder et al., 2016; Hughes et al., 2020; Rühlemann et al., 2021). For example, common genetic variation at the *NOD2* gene locus, a known IBD risk factor, was found to be significantly associated with the abundance of Enterobacteriaceae (Knights et al., 2014; Kurilshikov et al., 2017). Additional associations with variation in IBD-relevant genes were observed for other gut microbial features (Hu et al., 2021). In our own recent study of IBD families, we found 12 chromosomal regions to be genetically linked, at the genome-wide significance level, to different abundance-based microbiome traits (Sharma et al., 2022). These findings notwithstanding, the association between host genome and microbiome in IBD remains enigmatic which is illustrated, for example, by the observation of monozygotic twins who are discordant for UC and also have dissimilar microbiomes (Lepage et al., 2011).

The composition of the microbiome is the result of an interaction of its constituting taxa with both one another and the host. That these complex interactions also affect the health of the host is evidenced by the role, in many human diseases, of dysbiosis, i.e., an imbalance in the abundance of particular microbial community members (Layeghifard et al., 2017). In view of the clear link between intra-microbiome interaction and host health (Hansen et al., 2015; Hoffmann et al., 2016; Layeghifard et al., 2017), a microbial community-level approach should be taken when studying the biomedical relevance of certain changes in microbiome composition. To this end, microbiome data can efficiently be analyzed with graph-theoretical methods, including co-occurrence networks as a means to capture the correlation between species abundances (Tong et al., 2013; Layeghifard et al., 2017). Such networks allow a more systems-level view of microbial co-occurrence than the consideration of mere abundance values alone. Interestingly, in the specific context of IBD, it has been shown before that co-occurrence networks of human intestine 16S rRNA data contain subnetworks, termed “modules,” that are specifically associated with the disease (Tong et al., 2013). In addition, it has been shown that the type of gut microbiome dysbiosis often associated with diseases such as IBD can be characterized by correlation-based “co-abundance networks” as well (Chen et al., 2020).

Previous studies of the joint role of host genetics and gut microbiome in IBD etiology took a single-taxon level approach in unrelated individuals, assessing the abundance of individual species or genera for genotype associations at a genome-wide level. The present study, in contrast, involves classical genetic linkage analysis in IBD families and extends our previous single-taxon level investigations (Sharma et al., 2022) to the use of co-occurrence network modules. Based upon these modules, we defined novel quantitative traits capturing whole microbiome patterns in a single numerical figure to facilitate a multivariate, community-level search for genetic determinants of microbiome composition in IBD families. Our network-based approach also greatly alleviated the multiple testing problem affecting single-taxon level studies by the high dimensionality of the underlying microbiome data.

## 2 Materials and methods

### 2.1 Sample description

PopGen is a scientific biobank of residents of Schleswig-Holstein (Krawczak et al., 2006), the most northern federal state of Germany. In addition to various disease-specific sample collections, PopGen also maintains a control cohort from the general population (Ratjen et al., 2020). The population-based FoCus cohort is part of the Food Chain Plus (FoCus) study at Kiel, the capital of Schleswig-Holstein (Relling et al., 2018). The Kiel IBD kindred cohort (IBD-KC) is an ongoing nationwide prospective study of families of German IBD patients, coordinated from Kiel (Sharma et al., 2022). Stool samples were available for our study from 917 members of the PopGen control cohort, 1583 unrelated FoCus cohort members and 951 IBD-KC participants, both affected and unaffected. Since the construction of co-occurrence networks does not allow for any covariates of microbiome composition, we ensured that the different cohorts used in our study were generally comparable in terms of sex, age and BMI. To this end, plots of the age and sex distributions were visually assessed for sufficient similarity, and only individuals with a BMI <35 kg/m^2^ were included. We refrained from performing formal statistical tests because even small, and hence irrelevant, inter-group differences would have been declared statistically significant with the large sample sizes available to us.

### 2.2 16S RNA sequencing and data processing

Fecal samples of study participants were collected at home using standard stool collection tubes. Samples were mailed to the study center and stored at −80°C until processing. DNA was extracted from ∼200 mg of fecal material using QIAamp DNA stool mini-kits, automated on the QIAcube (QIAGEN). Subsequent 16S rRNA gene library preparation and sequencing were performed as previously described (Wang et al., 2016). In short, the V1-V2 region of the 16S rRNA gene was sequenced on the MiSeq platform using v3 chemistry for 2 × 300 bp paired-end reads (Illumina Inc., San Diego, CA, United States). Demultiplexing after sequencing was based upon the complete absence of mismatches in the barcode sequences. Data processing was performed with the DADA2 version 1.10 workflow for big datasets (https://benjjneb.github.io/dada2/bigdata.html), resulting in ASV abundance tables. Different sequencing runs were handled separately (see https://github.com/mruehlemann/ikmb_amplicon_processing/blob/master/dada2_16S_workflow.R for a V1-V2-adjusted workflow) and were only finally collapsed into a single abundance table per dataset, which then underwent chimaera filtering. ASVs were subjected to taxonomic annotation using the Bayesian classifier provided by DADA2 and the Ribosomal Database Project (RDP) version 16 release. The processing of microbiome data from fecal samples from the FoCus and PopGen cohorts has been described elsewhere (Rühlemann et al., 2021).

### 2.3 Single nucleotide polymorphism (SNP) genotyping

Genotyping was confined to IBD-KC samples and was carried out with the Global Screening Array-24 Multi Disease v2.0, following the Illumina Infinium HTS Assay Auto Workflow (Document #15045738v0). Subsequent steps, including QC of SNP genotypes, are described elsewhere (Sharma et al., 2022).

### 2.4 Data analysis

#### 2.4.1 Network construction and module detection

Microbiome networks were constructed from 16S rRNA data using pairwise Pearson correlation coefficients of microbial abundances to quantify “co-occurrence” (Tong et al., 2013). Each node of a microbiome co-occurrence network represents a genus, and the edge connecting two nodes (i.e., genera) reflects the strength of their correlation in abundance. We computed correlation matrices at the genus level separately for the PopGen and FoCus samples using FastSpar (Watts et al., 2019), which is a C++ implementation of the Sparse Correlations for Compositional data (SparCC) algorithm (Friedman and Alm, 2012). Only genera present in both data sets were used for network construction to allow meaningful inter-cohort comparison of the results. Worthy of note, SparCC log-ratio-transforms microbial abundance values before estimating the Pearson correlation coefficient for pairs of genera. We then used R package WGCNA (Weighted Correlation Network Analysis) (Langfelder and Horvath, 2008; 2012) to construct weighted signed adjacency matrices from Pearson correlation coefficients as a basis of subsequent hierarchical clustering of genera by way of average linkage. Finally, modules were identified in the resulting dendrograms of the PopGen and FoCus samples using the dynamic tree cut algorithm (Langfelder et al., 2008), setting the minimum module size to 10 genera. There was no upper limit for the module size. A balance between generating too large and too small modules was achieved by setting the *deepSplit* parameter to 2. All other parameters were set to default. Notably, this workflow had been followed before in a network-based gut microbiome analysis of unrelated IBD patients (Tong et al., 2013).

#### 2.4.2 Network module preservation

To investigate the robustness of the microbiome co-occurrence networks generated in different cohorts, we first investigated the extent to which the network modules identified in PopGen were preserved in FoCus, and *vice versa*. To this end, we employed an R implementation of the NetRep workflow (Ritchie et al., 2016), which entails a permutation-based assessment of the preservation of modules by seven different statistical tests of the null hypothesis of no module preservation. NetRep recalculates each test statistic after randomly permuting node labels, i.e., after shuffling the module affiliation of genera. For sparse microbiome data, it has been suggested to use only four of the seven statistics in question, namely coherence, average node contribution, density of the correlation structure, and average edge weight (Ritchie et al., 2016). A module detected in one dataset was considered “strongly preserved” in the other dataset if *p* < 0.05/4 = 0.012 for all four statistics (i.e., significant after Bonferroni correction), and “weakly preserved” if *p* < 0.012 for at least one, but not all statistics. This way, we assessed the level of module preservation between the PopGen and FoCus samples twice, considering one cohort as the discovery dataset and the other as the test dataset. For each analysis, the number of permutations was set to 10,000.

#### 2.4.3 Module-based quantitative trait (QT) definition in the IBD-KC dataset

Since the network modules identified in PopGen and FoCus showed convincing evidence for preservation across cohorts, we combined the two datasets into a single “control” dataset. In so doing, we kept only genera that were present in both datasets. Following network creation and module identification as described above, the genera constituting a network module in controls were next used to highlight the respective module in the IBD-KC dataset. Principal component analysis (PCA) of the microbial abundance values was then performed in the control dataset separately for each module, and the resulting loadings were used to project each IBD-KC sample onto the corresponding, module-specific first principal component (PC1). In subsequent linkage analyses of the IBD-KC families, the PC1 values served as quantitative traits (QT) to measure the level of co-occurrence of the genera constituting a given microbial network module in the control dataset.

Differences in terms of a given microbial module QT between the control dataset and either the affected or the unaffected part of IBD-KC were assessed for statistical significance using a Mann-Whitney *U* test. For each test, only one randomly selected individual per IBD-KC family was included to account for familial correlations. By contrast, a Wilcoxon signed-rank test was used to evaluate microbial module QT differences between affected and unaffected individuals within IBD-KC, considering one randomly selected pair of affected and unaffected members per family. Group-wise medians and quartiles are reported together with *p*-values from inter-group comparisons.

#### 2.4.4 Heritability analysis

A pedigree-based estimate of the heritability (h^2^) of each microbial module QT was obtained using the variance components method as implemented in MERLIN v1.1.2 (Abecasis et al., 2002). Sex, smoking status, IBD status, BMI and age were included as covariates in this and all other statistical analyses.

#### 2.4.5 Genome-wide QTL linkage analysis

We performed genome-wide multipoint QT locus (QTL) linkage analysis with MERLIN-REGRESS v1.1.2 to detect chromosomal regions linked to one or more of the microbial module QTs in IBD-KC families. The software is an implementation of the regression-based method proposed by Sham et al. (2002), which relates the estimated local level of identical-by-descent sharing of SNP haplotypes to both the squared sum and squared difference of the QT of interest. Individual IBD disease status was not accounted for in the analysis. We report LOD (“logarithm of odds”) scores as the key test statistic, with LOD scores >3 corresponding to significant evidence for linkage at the genome-wide level (Morton, 1998). As described before (Sharma et al., 2022), all subsequent analyses were confined to the so-called “2-units support intervals,” defined as the chromosomal regions around significant linkage peaks in which the LOD scores of other SNPs deviate by less than two units from the maximum LOD score (Ott, 1999). All genes located within 2-units support intervals were identified and a complete list of associated Gene Ontology (GO) terms was created for each protein-coding gene to pinpoint biologically plausible candidates for the causation of the linkage signals.

#### 2.4.6 Association analysis within linkage regions

We also performed family-based association analyses between microbial module QTs and individual SNPs from the QT-linked 2-units support intervals. Statistical testing of the SNP associations was performed using the WISARD workbench (Lee et al., 2018), which is an implementation of the genome-wide efficient mixed-model association algorithm (GEMMA) to fit a linear mixed model under the inclusion of a kinship matrix to account for familial relationships (Zhou and Stephens, 2012). All SNPs within the 2-units support intervals were looked up in previously published results of GWAS of IBD, UC or CD, drawing upon the GWAS Catalog (Version 1.0, downloaded on 9 July 2022 as gwas_catalog_v1.0-associations_e106_r2022-07-09.tsv).

#### 2.4.7 Comparison of module-based and single-taxon linkage analysis

To assess whether the network-based approach used in the present study revealed any relevant linkage signals beyond those already identified in our previous, single-taxon level analysis (Sharma et al., 2022), we identified all genera considered in the latter study that were included in at least one module with a significant linkage signal in the present study. For the 2-units support interval of a QT-specific linkage signal, we then recorded the average maximum LOD score from our previous study, taken over all genera constituting the respective module. For each linkage signal, this was repeated for 10,000 genomic regions of the same genetic length as the 2-units support interval in question, but picked at random from the human genome. The corresponding *p*-value was then estimated by the relative number of times the average maximum LOD score in the randomly picked regions was greater than the original one.

## 3 Results

### 3.1 Cohort characteristics

Healthy controls in our study were members of the population-based PopGen and FoCus cohorts with a BMI <35 kg/m^2^. While the age distribution was similar in the two control samples, the PopGen controls were found to be somewhat enriched with male samples (Supplementary Figure S1). For our QTL linkage analysis, we used genotype, phenotype and microbiome data from the IBD-KC, comprising 256 families. No relevant differences in terms of potential confounders such as age and sex were observed between affected and non-affected IBD-KC members. Overall, our study drew upon microbiome and supplementary data from 824 to 1049 healthy PopGen and FoCus members, respectively, and from 703 IBD-KC participants (Table 1). There were 427, 526 and 504 microbial genera present in the PopGen, FoCus and IBD-KC cohorts, respectively.

**TABLE 1:** Demographic characteristics [median (1st quartile, 3rd quartile) or number (%)] of study samples.

	PopGen controls	FoCus	IBD-KC	
			IBD	Non-IBD
Total (n)	824	1049	294	409
Age (years)	62 [54, 71]	54 [44, 65]	41.9 [30.2, 53.0]	42.6 [26.0, 58.2]
Body mass index (kg/m^2^)	26.5 [23.9, 29.1]	25.6 [22.2, 28.7]	23.9 [20.9, 26.4]	24.8 [21.4, 27.3]
Female [n, (%)]	344 [41.7]	617 [58.8]	182 [61.9]	231 [56.5]
Ever smoker [n, (%)]	506 [61.4]	629 [59.9]	29 [9.2]	50 [12.2]

### 3.2 Network construction and module detection

**FIGURE 1:**
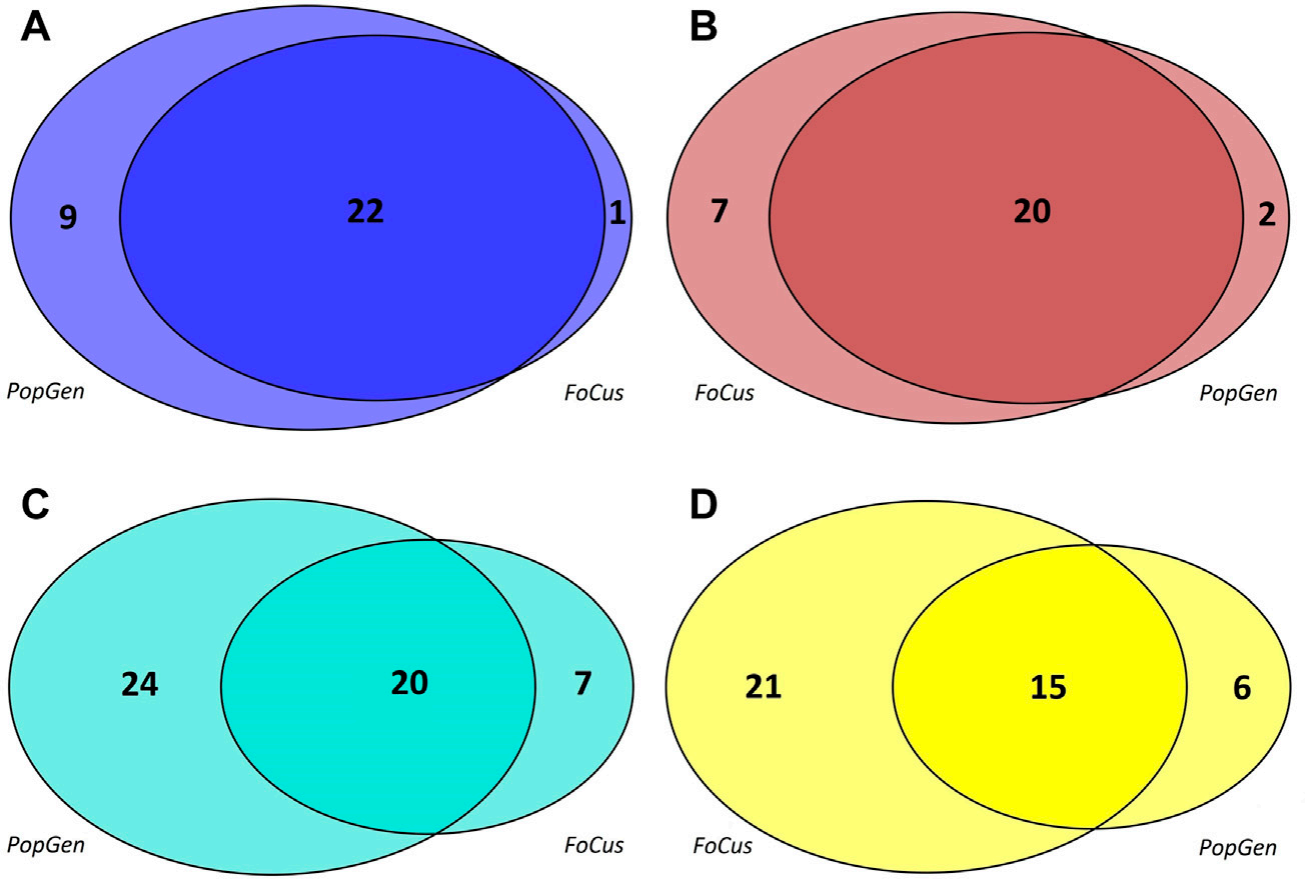
Modules detected, by WGCNA, in the microbial co-occurrence networks of the PopGen and FoCus samples. The size of each circle reflects the number of constituent genera of the corresponding module; each circle segment contains the number of genera included. For consistency, the original WGCNA coloring was modified based upon the maximum pairwise overlap between modules. **(A)**: blue module, **(B)**: brown module, **(C)**: turqoise module, **(D)**: yellow module.

Co-occurrence networks of microbial genera were generated separately for the PopGen and FoCus samples, based upon the 362 genera that were present in both cohorts. Four microbial modules each were detected in PopGen and FoCus, respectively (see Supplementary Table S1 for the constituent genera, see Figure 1 for the respective module sizes). The blue module from PopGen and FoCus contained 31 and 23 genera, respectively, with an overlap of 22 genera, “brown” contained 22 and 27 genera (overlap: 20), “turquoise” contained 44 and 27 genera (overlap: 20), and “yellow” contained 21 and 36 genera (overlap: 15). Since WGCNA uses arbitrary color schemes to label modules, we chose to relabel the modules detected in PopGen and FoCus based upon maximum pairwise overlap. We deliberately abstained from using functional or taxonomical characteristics to label modules in order to avoid any short-sighted interpretation of our linkage findings.

### 3.3 Network module preservation

Whether modules detected in the PopGen samples were preserved in FoCus, and *vice versa*, was assessed with NetRep, adopting a permutation-based testing approach that employs four different preservation statistics relevant for microbiome data. We assessed module preservation twice, switching the discovery and test dataset status between FoCus and PopGen (Supplementary Table S2). Three of the four modules detected in FoCus (original color labels “blue,” “turquoise,” “yellow”) were strongly preserved in PopGen whilst all PopGen modules were only “weakly” preserved in FoCus (see Methods). Nevertheless, for PopGen modules “blue” and “brown,” three of the four preservation statistics (all except coherence) were significant as well (i.e., *p* < 0.012).

### 3.4 Module-based quantitative trait definition in the IBD-KC dataset

**FIGURE 2:**
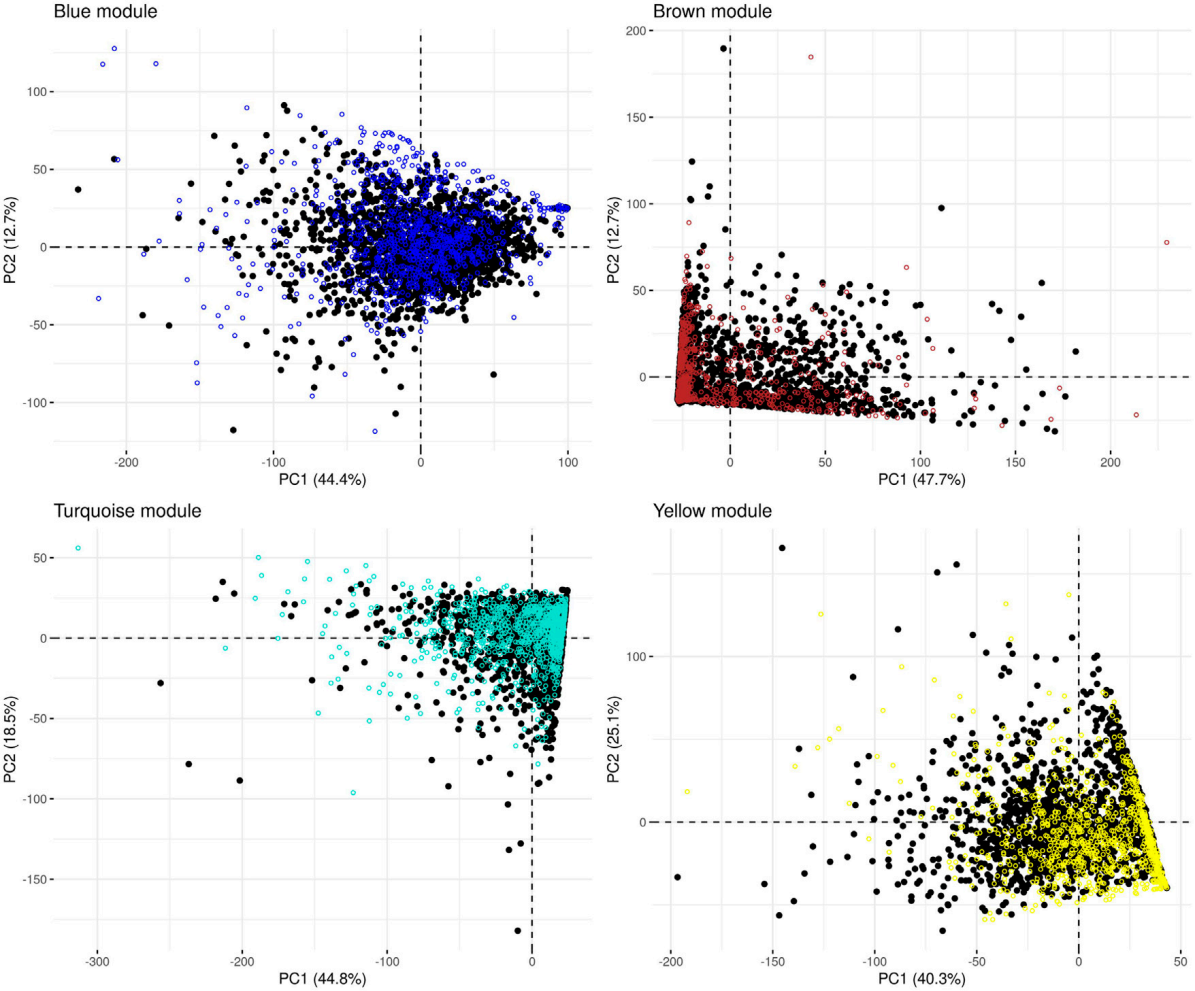
Principal component analysis of the abundance of microbial co-occurrence network modules in the combined control dataset. Black dots: control samples; colored dots: projected IBD-KC samples. The percentage of variance explained by a given PC is shown in brackets in the respective axis legend.

In the microbiome co-occurrence network generated from the (combined) control dataset, we detected four microbial modules that were also labelled “blue,” “brown,” “turquoise” and “yellow” by the WGCNA software (Supplementary Table S3). We re-identified these modules in the IBD-KC data through consideration of the respective constituting genera. In each module, the majority of genera belonged to phylum *Firmicutes* (blue: 43%, brown: 50%, turquoise: 60%, yellow: 76%). Next, we performed PCA of the abundance of the module-constituting genera and, from the results of the PCA, calculated the microbial module QT of each IBD-KC sample by projecting the sample onto the respective PC1 (Figure 2). Notably, all modules were found to contain genera characterized by a high percentage of zero abundance values (Supplementary Figure S2). While individuals with zero abundance for all constituting genera of a module cannot be separated by PCA, those with zero abundance for most, but not all, genera may end up on a straight line in the PCA plot (see brown, turquoise and yellow modules in Figure 2). However, since all module-specific PC1 derived here explained at least 40% of the overall variability in abundance in the combined control dataset, and since the projection of the IBD-KC samples (colored dots in Figure 2) showed substantial variation for all modules, we considered the resulting QTs suitable for subsequent linkage analysis.

**FIGURE 3:**
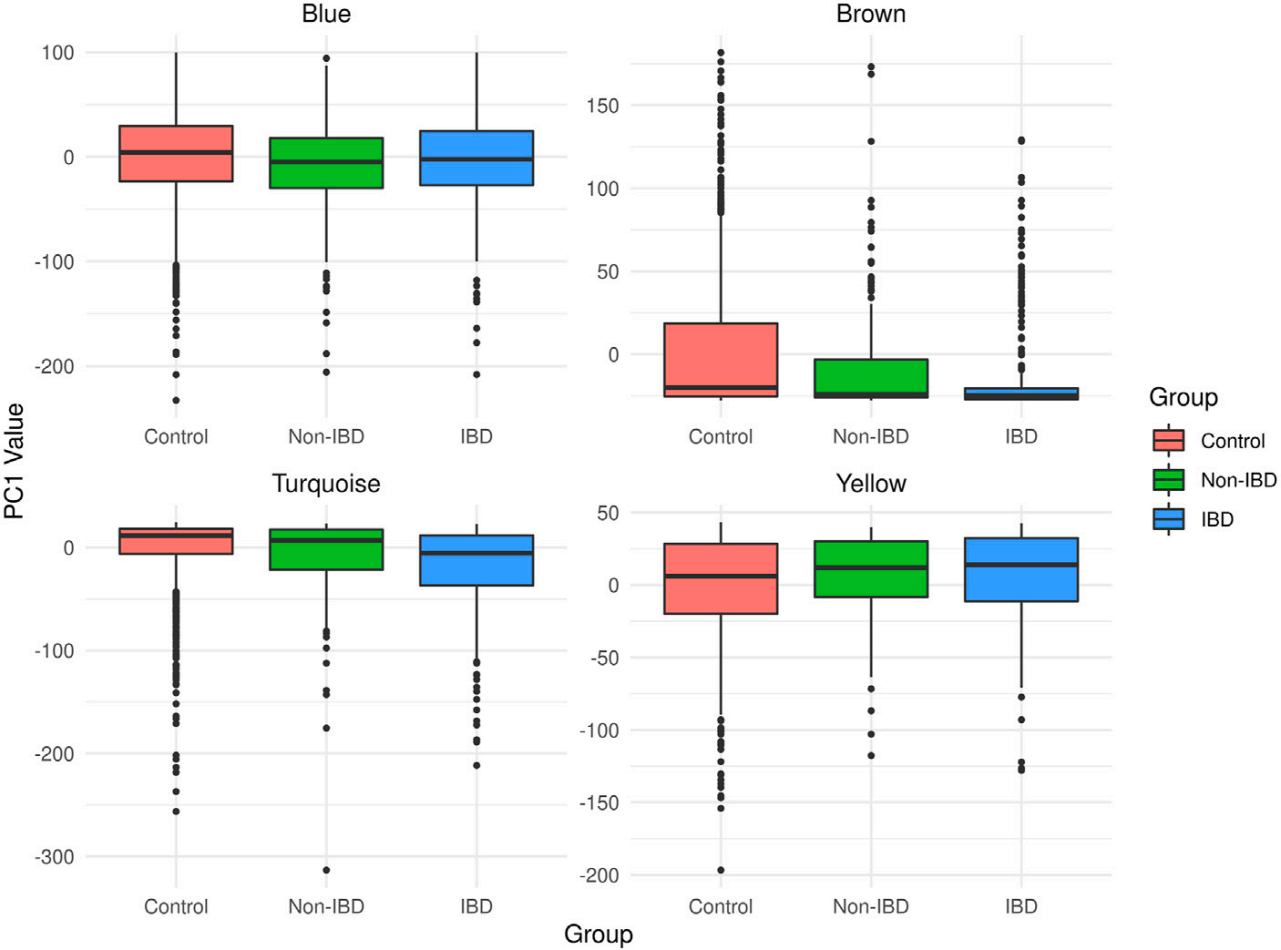
Boxplots of the microbial module QT distributions in the combined control cohort as well as in affected (IBD) and unaffected (non-IBD) IBD-KC individuals.

The microbial module QTs observed in the different study groups are depicted in Figure 3. Statistically significant inter-group differences were observed for most modules (see Table 2). In particular, the brown, turquoise and yellow module QTs differed significantly between IBD patients and unrelated healthy controls while the brown and turquoise module QTs also differed between IBD patients and their unaffected relatives. These results suggest that the alteration of at least three of the four microbial modules identified in our controls is relevant to IBD.

**TABLE 2:** Microbial module QTs in the combined control cohort as well as in affected (IBD) and unaffected (non-IBD) IBD-KC individuals. Shown are group-specific medians [1st quartile, 3rd quartile] and *p*-values from inter-group comparisons.

	Median [1^st^ quartile, 3^rd^ quartile]			*p*-value		
Module	CT	Non-IBD	IBD	CT vs. IBD	CT vs. non-IBD	IBD vs. non-IBD
Blue	4.16 [−23.42, 29.49]	−4.92 [−29.90, 17.71]	−2.41 [−25.93, 23.82]	0.14	0.0004	0.1
Brown	−20.02 [−25.25, 18.58]	−24.05 [−25.96, -3.24]	−24.95 [−27.19, -21.29]	<0.0001	<0.0001	0.0061
Turquoise	11.43 [−6.32, 18.07]	6.87 [−21.64, 17.42]	−7.01 [−37.17, 12.49]	<0.0001	00039	0.0009
Yellow	6.04 [−19.94, 28.45]	11.97 [−8.38, 30.17]	13.35 [−13.25, 32.21]	0.005	0.0032	0.56

### 3.5 Heritability analysis of microbial module QTs

The covariate-adjusted, pedigree-based estimate of the heritability (h^2^) of the corresponding microbial module QT equaled 6.4% for the brown module, 14.1% for the turquoise module, 16.7% for the yellow module and 32.0% for the blue module. Notably, the brown module had the lowest heritability in the IBD-KC families, which may be related to the fact that all constituent genera had an abundance of zero in >20% of the CT individuals (Supplementary Figure S2) and that the variability of the QT was the lowest of all modules (Figure 3).

### 3.6 Genome-wide module QTL linkage analysis

**FIGURE 4:**
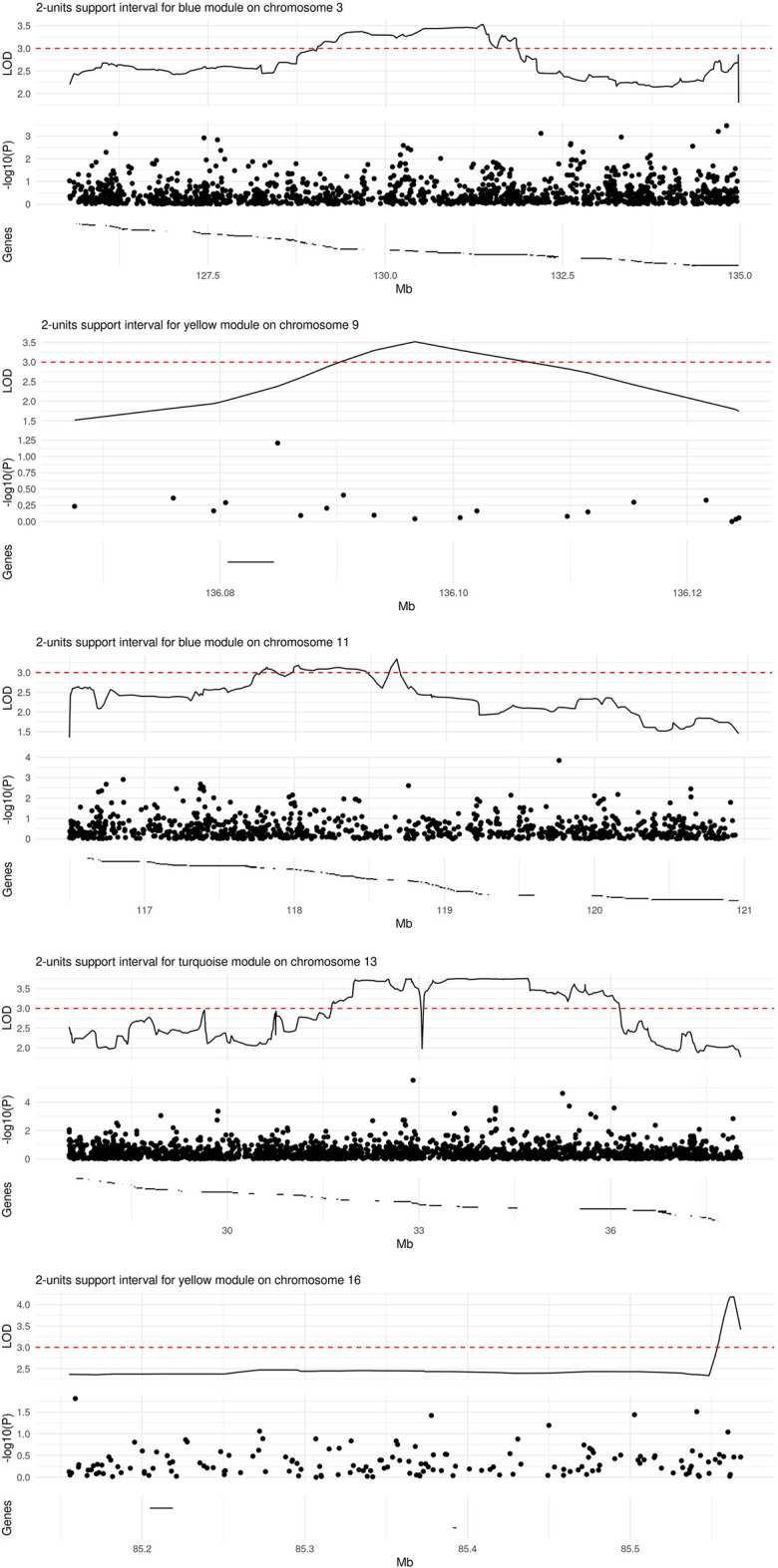
Regions of significant linkage with microbial module QTs. The locus zoom plots span the 2-units support intervals of a given genome-wide significant linkage signal. Depicted are the LOD score (top panel), *p*-value (on the log10 scale) from a SNP-wise association analysis (central panel), and the location of protein-coding genes (bottom panel). The range of a given gene is marked by a horizontal line; region-wise lists of gene names (from left to right) are provided in Supplementary Table S4.

We observed significant evidence for linkage at genome-wide level (i.e., LOD score >3) between five chromosomal regions and three microbial module QTs, namely blue [chr3. maximum LOD score (max LOD) 3.53; chr11, max LOD 3.34], yellow (chr9, max LOD 3.52; chr16, max LOD 4.18) and turquoise (chr13, max LOD 3.76) (Table 3; Figure 4). No significant linkage was observed for the QT corresponding to the brown module, possibly due to its low heritability. A summary of the results of the genome-wide QTL linkage analysis is provided in Supplementary Figure S3. Protein-coding genes located within the 2-units support intervals of the peak linkage signals are listed in Supplementary Table S4 whilst the GO terms associated with these genes are provided in Supplementary Table S5.

**TABLE 3:** Genome-wide QTL linkage analysis of microbial module QTs in IBD families. For each significantly linked locus, the 2-units support interval demarcates the chromosomal region in which the LOD score differs by less than two units from the maximum LOD score. The number, n, of genes in each region was determined using biomaRt v.2.44.4 (Durinck et al., 2009). Mb: Mega base pairs; cM: centi-Morgan.

Module	Chromosome	2-Units support interval	Physical (genetic) size	Genes (n)
Blue	3	rs2979948—rs116052381	9.41 Mb (10.79 cM)	209
Yellow	9	rs75071805—rs9411370	0.06 Mb (0.13 cM)	2
Blue	11	rs76517460—rs11600109	4.44 Mb (6.69 cM)	148
Turquoise	13	rs11618775—rs117621393	10.51 Mb (19.98 cM)	158
Yellow	16	rs57083127—rs9927472	0.41 Mb (1.20 cM)	6

### 3.7 Association analysis within linkage regions

Genetic association analyses within the 2-units support intervals linked to different microbial module QTs comprised a variable number of SNPs (Figure 4): 1422 for blue/chr3, 19 for yellow/chr9, 962 for blue/chr11, 2138 for turquoise/chr13, and 157 for yellow/chr16. None of the SNPs was significantly associated with the corresponding module QT after Bonferroni correction for multiple testing. A list of all nominally significant SNP-QT associations is provided in Supplementary Table S6. Three of the SNPs within the 2-units support intervals (rs17085007, rs12585310, and rs630923) have been implicated previously in IBD, UC or CD by GWAS (Liu et al., 2015; Ellinghaus et al., 2016; de Lange et al., 2017; for details see Supplementary Table S7).

### 3.8 Comparison of module-based and single-taxon level linkage analysis

In order to assess the advantages of a network-based approach compared to our previous single-taxon level analysis, we identified all genera included in a given module with a significant QT linkage signal in the present study and recorded the genus-specific maximum LOD scores, in the corresponding 2-units support interval, that were attained in our previous study. We then adopted a randomization-based approach to determine the *p*-value of each average maximum LOD score, taken over the genera included in a given module (see Methods). None of the observed average maximum LOD scores was significantly higher than its random expectation (Table 4), implying that all network-based linkage signals detected in the present study were indeed novel and not noticeable in our previous single-taxon level analysis of the same data.

**TABLE 4:** Comparison of linkage signals from module-based and single-taxon level linkage analysis.

Module (chromosome)	Module-based max LOD	Average single-taxon max LOD	*p*-value (average single-taxon max LOD)
Blue (chr 3)	3.53	0.32	0.61
Blue (chr 11)	3.34	0.04	0.95
Turquoise (chr 13)	3.76	0.70	0.24
Yellow (chr 9)	3.52	0.11	0.21
Yellow (chr 16)	4.18	0.33	0.13

## 4 Discussion

We investigated the link between host genetics and gut microbiome composition in the specific context of inflammatory bowel disease (IBD) using novel microbiome-related quantitative traits (QTs) that were derived from the modules identified in microbial co-occurrence networks. More specifically, co-occurrence networks based upon 16S RNA sequence data were first created separately for two non-overlapping cohorts of healthy controls, followed by an assessment of the mutual preservation of the modules included in the two networks. In view of the results, the two control datasets were combined into one and the modules identified in the joint co-occurrence network formed the basis of the definition, by way of principal component analysis, of QTs in families of IBD patients. These QTs somehow quantify the level of coherence of individual 16S RNA profiles with general patterns of microbial co-occurrence in the guts of healthy controls. Therefore, we estimated the heritability of each microbial module QT and performed a genome-wide QTL linkage analysis, in IBD-KC families, to search for its genetic determinants. To our knowledge, our study is the first to use QTs derived from modules of microbial co-occurrence networks as phenotypes in a genetic linkage analysis.

As indicated above, our analyses revealed a high level of module preservation between the two cohorts of German healthy controls underlying the present study. This not only suggests that microbiome composition may be a comparatively stable biological trait in healthy individuals but also justified the combination of the two control cohorts into one. Although many of the module-constituting genera were only present in a small proportion of individuals, their co-occurrence was still essential for the formation of the respective module. Estimates of the heritability of the microbial module QTs derived from the control dataset were consistent with previous reports (Goodrich et al., 2016; Turpin et al., 2016; Lim et al., 2017) that microbial traits have lower heritability than other (heritable) human traits. Notably, the final QTL linkage analyses led to the identification of five different chromosomal regions individually linked to one of three of the microbial modules QTs. The QT corresponding to the brown module, which contained only genera of very low abundance, had the lowest heritability of all and did not show any evidence of linkage.

Although challenging (Awany et al., 2019), GWAS of microbial traits have led to the detection of a number of host genome-microbiome associations (Bonder et al., 2016; Hughes et al., 2020; Rühlemann et al., 2021). In the specific context of IBD, *NOD2* gene variation was found to be related to the abundance of specific microbial taxa (Kurilshikov et al., 2017). Taken together with the fact that the microbiomes of IBD patients were consistently found to be altered, compared to healthy controls, these GWAS results pointed towards a joint role of microbiome and host genetics in shaping the IBD phenotype. However, both the present and our previous study were carried out under the premise that the causal connection between microbiome and disease depends upon additional genes, other than known IBD risk genes. These genes were searched for by way of classical (pedigree-based) linkage analysis. Similar to comparable studies in the field, we initially only looked at individual taxa so that higher-level associations between host genetics and gut microbiome in IBD etiology may have been overlooked. Therefore, we expanded our single-taxon level search for genetic modifiers of microbiome composition in IBD families to the linkage analysis of quantitative traits reflecting the coherence of a given gut microbiome with general patterns of co-abundance. In addition, we investigated whether the observed linkage signals were novel and had not been evident already at single-taxon level. The results indicate that our module-based analysis, addressing the joint effect of a group of correlated microbes, was indeed valuable in that it yielded five novel microbiome-related linkage signals of genome-wide significance.

Although not formally accounting for the IBD status of single individuals, our linkage analysis nevertheless aimed at uncovering genetic determinants of microbial composition in the specific context of IBD families. This approach was motivated by the plausible assumption that genetic factors that influence microbiome composition are not necessarily doing so in the same way and with the same result in all humans. Instead, it is likely that the relationships between genetic variants and microbial traits depend upon additional environmental and genetic co-factors. Therefore, a link between genetics and the microbiome that is important against the specific environmental and genetic background of an IBD family may simply have been overlooked in less specific, particularly population-wide, studies. Moreover, the fact that three of the four microbial QTs analyzed for linkage also differed significantly between IBD patients and unaffected individuals emphasizes the likely connection between these phenotypes and the disease. Whether genetic variation in the identified linkage regions influences the QTs differently in affected and non-affected individuals, i.e., whether there is statistical interaction between genotype and IBD status with regard to gut microbiome composition, requires additional research in larger family or population-based studies of IBD.

Our study was performed in Germany and it has been observed that human gut microbiomes of geographically distinct populations show wide variation in terms of their composition (Porras et al., 2021). However, to the best of our knowledge, the preservation of co-occurrence network-derived modules across populations and ethnicities has not been investigated in much detail yet. Moreover, it may be argued that the genetic basis of the module-derived QTs studied here is likely to be widely overlapping between populations, even if their statistical distributions differ. In addition, genetic linkage depends much less upon population history than the genotype-phenotype associations targeted by GWAS. This is because linkage analysis considers the co-segregation of genotypes and phenotypes in families (usually for no more than one or two generations), and its results hence depend upon recombination frequency (or inter-locus genetic distance). By contrast, GWAS exploit statistical associations at the population level that result from shared ancestry of phenotypically similar individuals over long periods of historical time. This notwithstanding, future studies of the genetic basis of microbial features should aim at including cohorts with diverse geographic and ethnic backgrounds so as to pinpoint both common and population-specific etiological factors.

Another aspect worth noting is that our QT linkage analysis addressed taxonomical co-occurrence, drawing upon 16S rRNA data. Of course, this type of inter-genera relationship differs from correlation at the functional level which could be captured, for example, by shotgun metagenomics data. Since functional and taxonomical relatedness are evolutionarily connected, the two are not expected to deviate greatly. However, since microbiome function likely contributes to host-microbiome interaction at least as much as microbial taxonomy, the former should explicitly be evaluated for its connection to host genetic factors as well. Unfortunately, we did not have the necessary data to do so in the present study.

In the combined control dataset underlying the present study, we identified four microbial co-occurrence network modules that were color-labeled automatically by the software used. Interestingly, six genera present in module “turquoise,” namely *Staphylococcus*, *Porphyromonas*, *Fusobacterium*, *Lactobacillus*, *Enterococcus* and *Corynebacterium*, were also present in a network module related before to relapsing refractory IBD (Yilmaz et al., 2019). This finding lends additional support to the idea that microbial modules can be transformed into QTs that segregate in IBD families, and that are therefore worth studying by means of linkage analysis. Moreover, the turquoise module was dominated by genera belonging to taxonomic family Lachnospiraceae (>20%), members of which are known producers of short-chain fatty acids and a reduction of which has been related before to microbial dysbiosis in IBD patients in particular (Deleu et al., 2021).

Our genome-wide QT linkage analysis provided evidence for linkage between five different chromosomal regions and one of three microbial module QTs. Worthy of note, SNPs rs17085007 and rs12585310, which are located in the chr13 region (Figure 4D) linked to the turquoise module QT, as well as SNP rs630923 located in chr11 region (Figure 4C) linked to the blue module QT, have been reported before to be associated with IBD or one of its subtypes (Liu et al., 2015; Ellinghaus et al., 2016; de Lange et al., 2017). Of these SNPs, rs17085007 and rs12585310 map to the regulatory region of the *RPS21P8* (ribosomal protein S21 pseudogene 8) and *FGFR1OP2P1* (FGFR1 oncogene partner 2 pseudogene 1) genes while rs630923 maps to the regulatory region of the *CXCR5* (C-X-C motif chemokine receptor 5) gene, according to the GWAS Catalog (Buniello et al., 2019). This physical overlap between known IBD risk SNPs and genomic regions linked to microbiome-derived QTs adds further evidence to the disease relevance of our findings, particularly in view of the significant difference of the turquoise module QT between IBD patients and unaffected individuals.

Several other genes in the linked regions have been shown before to be connected to inflammation or to a response to bacterial infection, or their expression level has been associated with specific IBD characteristics. Thus, the *TF* (transferrin), *RAB7A* (member RAS oncogene family) and *MGLL* (monoglyceride lipase) genes, located in the chr3 region linked to the blue module QT, are connected to Gene Ontology (GO) terms “response to bacterium,” “antibacterial humoral response,” “inflammatory response” or “regulation of inflammatory response” (Ashburner et al., 2000; The Gene Ontology Consortium, 2021). Interestingly, increased expression of the *MGLL* gene has been observed before in intestinal mucosal biopsies from CD patients (Hryhorowicz et al., 2021). On the other hand, *TF* levels were not only found to be decreased in blood samples from IBD patients but were also negatively correlated with disease activity scores in active CD and UC (Matusiewicz et al., 2017).

Of the genes located in the chr11 region linked to the blue module QT, the *NLRX1* (NLR family member X1), *APOA1* (apolipoprotein A1) and *CBL* (Cbl proto-oncogene) genes are connected to GO terms “negative regulation of inflammatory response” or “entry of bacterium into host cell” while the *KL* (klotho), *HMGB1* (high mobility group box 1) and *ALOX5AP* (arachidonate 5-lipoxygenase activating protein) genes are connected to “acute inflammatory response,” “inflammatory response,” “inflammatory response to antigenic stimulus,” “leukotriene production involved in inflammatory response” or “positive regulation of acute inflammatory response”. Notably, *APOA1* expression was found to be reduced in ileal samples from treatment-naïve CD patients that showed reduced Firmicutes abundance (Haberman et al., 2014). The *NLRX1* gene is a known anti-inflammatory gene that protects against IBD in mouse models, and *NLRX1* ^−/−^ mice were reported to exhibit depletion of butyrate-producing microbial taxa like *Faecalibacterium* (Leber et al., 2018). Expression of the *ALOX5AP* gene was shown before to be downregulated in CD patients while renal expression of the *KL* gene was shown to be reduced in mice models of IBD (Thurston et al., 2010). Finally, fecal levels of the *HMGB1* gene product are a biomarker of intestinal inflammation that correlates with fecal calprotectin in IBD patients (Palone et al., 2016). Undoubtedly, these earlier reports lend additional support to the biological plausibility of our microbial network-based linkage findings in IBD families. A complete list of the GO terms associated with genes located in the 2-units support interval of linkage identified in our study is provided in Supplementary Table S5.

In conclusion, the present study has been the first to use microbial co-occurrence networks as the basis of defining quantitative traits for genetic linkage analysis in IBD families. All five linkage regions detected with this approach had not been uncovered by a previous, single-taxon level linkage study of the same data, and hence are likely to harbor hitherto unknown genetic drivers of microbiome composition in the specific context of IBD. Our study thus also illustrates the benefit of using microbial networks to capture and summarize community-level interactions in relation to a given disease, and of employing the results in subsequent family-based genetic analyses. Such advanced studies of microbial abundance data bear great potential to expand our understanding of the joint role of the host genome and the microbiome in shaping disease phenotypes.

## Data Availability

The data analyzed in this study is subject to the following licenses/restrictions: Data can be obtained through application to the PopGen 2.0 biobank. Requests to access these datasets should be directed to https://portal.popgen.de/.

## References

[B1] Abecasis G. R. Cherny S. S. Cookson W. O. Cardon L. R. (2002). Merlin—Rapid analysis of dense genetic maps using sparse gene flow trees. Nat. Genet. 30, 97–101. 10.1038/ng786 11731797

[B2] Alatab S. Sepanlou S. G. Ikuta K. Vahedi H. Bisignano C. Safiri S. (2020). The global, regional, and national burden of inflammatory bowel disease in 195 countries and territories, 1990–2017: A systematic analysis for the global burden of disease study 2017. Lancet Gastroenterology Hepatology 5, 17–30. 10.1016/S2468-1253(19)30333-4 31648971 PMC7026709

[B3] Ashburner M. Ball C. A. Blake J. A. Botstein D. Butler H. Cherry J. M. (2000). Gene ontology: Tool for the unification of biology. The gene Ontology Consortium. Nat. Genet. 25, 25–29. 10.1038/75556 10802651 PMC3037419

[B4] Awany D. Allali I. Dalvie S. Hemmings S. Mwaikono K. S. Thomford N. E. (2019). Host and microbiome genome-wide association studies: Current state and challenges. Front. Genet. 9, 637. 10.3389/fgene.2018.00637 30723493 PMC6349833

[B5] Bonder M. J. Kurilshikov A. Tigchelaar E. F. Mujagic Z. Imhann F. Vila A. V. (2016). The effect of host genetics on the gut microbiome. Nat. Genet. 48, 1407–1412. 10.1038/ng.3663 27694959

[B6] Buniello A. MacArthur J. A. L. Cerezo M. Harris L. W. Hayhurst J. Malangone C. (2019). The NHGRI-EBI GWAS Catalog of published genome-wide association studies, targeted arrays and summary statistics 2019. Nucleic Acids Res. 47, D1005–D1012. 10.1093/nar/gky1120 30445434 PMC6323933

[B7] Caruso R. Lo B. C. Núñez G. (2020). Host–microbiota interactions in inflammatory bowel disease. Nat. Rev. Immunol. 20, 411–426. 10.1038/s41577-019-0268-7 32005980

[B8] Chen L. Collij V. Jaeger M. van den Munckhof I. C. L. Vich Vila A. Kurilshikov A. (2020). Gut microbial co-abundance networks show specificity in inflammatory bowel disease and obesity. Nat. Commun. 11, 4018. 10.1038/s41467-020-17840-y 32782301 PMC7419557

[B9] de Lange K. M. Moutsianas L. Lee J. C. Lamb C. A. Luo Y. Kennedy N. A. (2017). Genome-wide association study implicates immune activation of multiple integrin genes in inflammatory bowel disease. Nat. Genet. 49, 256–261. 10.1038/ng.3760 28067908 PMC5289481

[B10] Deleu S. Machiels K. Raes J. Verbeke K. Vermeire S. (2021). Short chain fatty acids and its producing organisms: An overlooked therapy for IBD? eBioMedicine 66, 103293. 10.1016/j.ebiom.2021.103293 33813134 PMC8047503

[B11] Durinck S. Spellman P. T. Birney E. Huber W. (2009). Mapping identifiers for the integration of genomic datasets with the R/Bioconductor package biomaRt. Nat. Protoc. 4, 1184–1191. 10.1038/nprot.2009.97 19617889 PMC3159387

[B12] Ellinghaus D. Jostins L. Spain S. L. Cortes A. Bethune J. Han B. (2016). Analysis of five chronic inflammatory diseases identifies 27 new associations and highlights disease-specific patterns at shared loci. Nat. Genet. 48, 510–518. 10.1038/ng.3528 26974007 PMC4848113

[B13] Friedman J. Alm E. J. (2012). Inferring correlation networks from genomic survey data. PLOS Comput. Biol. 8, e1002687. 10.1371/journal.pcbi.1002687 23028285 PMC3447976

[B14] Goodrich J. K. Davenport E. R. Beaumont M. Jackson M. A. Knight R. Ober C. (2016). Genetic determinants of the gut microbiome in UK Twins. Cell Host Microbe 19, 731–743. 10.1016/j.chom.2016.04.017 27173935 PMC4915943

[B15] Haberman Y. Tickle T. L. Dexheimer P. J. Kim M.-O. Tang D. Karns R. (2014). Pediatric Crohn disease patients exhibit specific ileal transcriptome and microbiome signature. J. Clin. Invest. 124, 3617–3633. 10.1172/JCI75436 25003194 PMC4109533

[B16] Hansen T. H. Gøbel R. J. Hansen T. Pedersen O. (2015). The gut microbiome in cardio-metabolic health. Genome Med. 7, 33. 10.1186/s13073-015-0157-z 25825594 PMC4378584

[B17] Hoffmann A. R. Proctor L. M. Surette M. G. Suchodolski J. S. Rodrigues HoffmAnn A. (2016). The microbiome: The trillions of microorganisms that maintain health and cause disease in humans and companion animals. Vet. Pathol. 53, 10–21. 10.1177/0300985815595517 26220947

[B18] Hryhorowicz S. Kaczmarek-Ryś M. Zielińska A. Scott R. J. Słomski R. Pławski A. (2021). Endocannabinoid system as a promising therapeutic target in inflammatory bowel disease – a systematic review. Front. Immunol. 12, 790803. 10.3389/fimmu.2021.790803 35003109 PMC8727741

[B19] Hu S. Vila A. V. Gacesa R. Collij V. Stevens C. Fu J. M. (2021). Whole exome sequencing analyses reveal gene–microbiota interactions in the context of IBD. Gut 70, 285–296. 10.1136/gutjnl-2019-319706 32651235 PMC7815889

[B20] Hughes D. A. Bacigalupe R. Wang J. Rühlemann M. C. Tito R. Y. Falony G. (2020). Genome-wide associations of human gut microbiome variation and implications for causal inference analyses. Nat. Microbiol. 5, 1079–1087. 10.1038/s41564-020-0743-8 32572223 PMC7610462

[B21] Imhann F. Vila A. V. Bonder M. J. Fu J. Gevers D. Visschedijk M. C. (2018). Interplay of host genetics and gut microbiota underlying the onset and clinical presentation of inflammatory bowel disease. Gut 67, 108–119. 10.1136/gutjnl-2016-312135 27802154 PMC5699972

[B22] Knights D. Silverberg M. S. Weersma R. K. Gevers D. Dijkstra G. Huang H. (2014). Complex host genetics influence the microbiome in inflammatory bowel disease. Genome Med. 6, 107. 10.1186/s13073-014-0107-1 25587358 PMC4292994

[B23] Krawczak M. Nikolaus S. von Eberstein H. Croucher P. J. P. El Mokhtari N. E. Schreiber S. (2006). PopGen: Population-Based recruitment of patients and controls for the analysis of complex genotype-phenotype relationships. Public Health Genomics 9, 55–61. 10.1159/000090694 16490960

[B24] Kurilshikov A. Wijmenga C. Fu J. Zhernakova A. (2017). Host genetics and gut microbiome: Challenges and perspectives. Trends Immunol. 38, 633–647. 10.1016/j.it.2017.06.003 28669638

[B25] Langfelder P. Horvath S. Kipnis V. Midthune D. Freedman L. S. Carroll R. J. (2012). Intake_epis_food: An R function for fitting a bivariate nonlinear measurement error model to estimate usual and energy intake for episodically consumed foods. J. Stat. Softw. 46, 1–17. 10.18637/jss.v046.c03 22837731 PMC3403723

[B26] Langfelder P. Horvath S. (2008). Wgcna: an R package for weighted correlation network analysis. BMC Bioinforma. 9, 559. 10.1186/1471-2105-9-559 PMC2631488 19114008

[B27] Langfelder P. Zhang B. Horvath S. (2008). Defining clusters from a hierarchical cluster tree: The dynamic tree cut package for R. Bioinformatics 24, 719–720. 10.1093/bioinformatics/btm563 18024473

[B28] Lavoie S. Conway K. L. Lassen K. G. Jijon H. B. Pan H. Chun E. (2019). The Crohn’s disease polymorphism, ATG16L1 T300A, alters the gut microbiota and enhances the local Th1/Th17 response. eLife 8, e39982. 10.7554/eLife.39982 30666959 PMC6342529

[B29] Layeghifard M. Hwang D. M. Guttman D. S. (2017). Disentangling interactions in the microbiome: A network perspective. Trends Microbiol. 25, 217–228. 10.1016/j.tim.2016.11.008 27916383 PMC7172547

[B30] Leber A. Hontecillas R. Tubau-Juni N. Zoccoli-Rodriguez V. Abedi V. Bassaganya-Riera J. (2018). NLRX1 modulates immunometabolic mechanisms controlling the host–gut microbiota interactions during inflammatory bowel disease. Front. Immunol. 9, 363. 10.3389/fimmu.2018.00363 29535731 PMC5834749

[B31] Lee S. Choi S. Qiao D. Cho M. Silverman E. K. Park T. (2018). Wisard: Workbench for integrated superfast association studies for related datasets. BMC Med. Genomics 11, 39. 10.1186/s12920-018-0345-y 29697360 PMC5918457

[B32] Lepage P. Häsler R. Spehlmann M. E. Rehman A. Zvirbliene A. Begun A. (2011). Twin study indicates loss of interaction between microbiota and mucosa of patients with ulcerative colitis. Gastroenterology 141, 227–236. 10.1053/j.gastro.2011.04.011 21621540

[B33] Lim M. Y. You H. J. Yoon H. S. Kwon B. Lee J. Y. Lee S. (2017). The effect of heritability and host genetics on the gut microbiota and metabolic syndrome. Gut 66, 1031–1038. 10.1136/gutjnl-2015-311326 27053630

[B34] Liu J. Z. van Sommeren S. Huang H. Ng S. C. Alberts R. Takahashi A. (2015). Association analyses identify 38 susceptibility loci for inflammatory bowel disease and highlight shared genetic risk across populations. Nat. Genet. 47, 979–986. 10.1038/ng.3359 26192919 PMC4881818

[B35] Lloyd-Price J. Arze C. Ananthakrishnan A. N. Schirmer M. Avila-Pacheco J. Poon T. W. (2019). Multi-omics of the gut microbial ecosystem in inflammatory bowel diseases. Nature 569, 655–662. 10.1038/s41586-019-1237-9 31142855 PMC6650278

[B36] Matusiewicz M. Neubauer K. Lewandowska P. Gamian A. Krzystek-Korpacka M. (2017). Reduced transferrin levels in active inflammatory bowel disease. Biomed. Res. Int. 2017, 9541370. 10.1155/2017/9541370 29226154 PMC5684570

[B37] Morton N. E. (1998). Significance levels in complex inheritance. Am. J. Hum. Genet. 62, 690–697. 10.1086/301741 9497238 PMC1376937

[B38] Ott J. (1999). Analysis of human genetic linkage. 3rd ed. Baltimore and London: Johns Hopkins University Press.

[B39] Ott S. J. Musfeldt M. Wenderoth D. F. Hampe J. Brant O. Fölsch U. R. (2004). Reduction in diversity of the colonic mucosa associated bacterial microflora in patients with active inflammatory bowel disease. Gut 53, 685–693. 10.1136/gut.2003.025403 15082587 PMC1774050

[B40] Palone F. Vitali R. Cucchiara S. Mennini M. Armuzzi A. Pugliese D. (2016). Fecal HMGB1 reveals microscopic inflammation in adult and pediatric patients with inflammatory bowel disease in clinical and endoscopic remission. Inflamm. Bowel Dis. 22, 2886–2893. 10.1097/MIB.0000000000000938 27755215

[B41] Porras A. M. Shi Q. Zhou H. Callahan R. Montenegro-Bethancourt G. Solomons N. (2021). Geographic differences in gut microbiota composition impact susceptibility to enteric infection. Cell Rep. 36, 109457. 10.1016/j.celrep.2021.109457 34320343 PMC8333197

[B42] Ratjen I. Morze J. Enderle J. Both M. Borggrefe J. Müller H.-P. (2020). Adherence to a plant-based diet in relation to adipose tissue volumes and liver fat content. Am. J. Clin. Nutr. 112, 354–363. 10.1093/ajcn/nqaa119 32453423

[B43] Relling I. Akcay G. Fangmann D. Knappe C. Schulte D. M. Hartmann K. (2018). Role of wnt5a in metabolic inflammation in humans. J. Clin. Endocrinol. Metabolism 103, 4253–4264. 10.1210/jc.2018-01007 30137542

[B44] Ritchie S. C. Watts S. Fearnley L. G. Holt K. E. Abraham G. Inouye M. (2016). A scalable permutation approach reveals replication and preservation patterns of network modules in large datasets. Cell Syst. 3, 71–82. 10.1016/j.cels.2016.06.012 27467248

[B45] Rühlemann M. C. Hermes B. M. Bang C. Doms S. Moitinho-Silva L. Thingholm L. B. (2021). Genome-wide association study in 8, 956 German individuals identifies influence of ABO histo-blood groups on gut microbiome. Nat. Genet. 53, 147–155. 10.1038/s41588-020-00747-1 33462482

[B46] Sham P. C. Purcell S. Cherny S. S. Abecasis G. R. (2002). Powerful regression-based quantitative-trait linkage analysis of general pedigrees. Am. J. Hum. Genet. 71, 238–253. 10.1086/341560 12111667 PMC379157

[B47] Sharma A. Szymczak S. Rühlemann M. Freitag-Wolf S. Knecht C. Enderle J. (2022). Linkage analysis identifies novel genetic modifiers of microbiome traits in families with inflammatory bowel disease. Gut Microbes 14, 2024415. 10.1080/19490976.2021.2024415 35129060 PMC8820822

[B48] The Gene Ontology Consortium (2021). The gene ontology resource: Enriching a GOld mine. Nucleic Acids Res. 49, D325–D334. 10.1093/nar/gkaa1113 33290552 PMC7779012

[B49] Thurston R. D. Larmonier C. B. Majewski P. M. Ramalingam R. Midura-Kiela M. Laubitz D. (2010). Tumor necrosis factor and interferon-γ down-regulate klotho in mice with colitis. Gastroenterology 138, 1384–1394. e2. 10.1053/j.gastro.2009.12.002 20004202 PMC3454518

[B50] Tong M. Li X. Wegener Parfrey L. Roth B. Ippoliti A. Wei B. (2013). A modular organization of the human intestinal mucosal microbiota and its association with inflammatory bowel disease. PLoS One 8, e80702. 10.1371/journal.pone.0080702 24260458 PMC3834335

[B51] Turpin W. Espin-Garcia O. Xu W. Silverberg M. S. Kevans D. Smith M. I. (2016). Association of host genome with intestinal microbial composition in a large healthy cohort. Nat. Genet. 48, 1413–1417. 10.1038/ng.3693 27694960

[B52] Wang J. Thingholm L. B. Skiecevičienė J. Rausch P. Kummen M. Hov J. R. (2016). Genome-wide association analysis identifies variation in vitamin D receptor and other host factors influencing the gut microbiota. Nat. Genet. 48, 1396–1406. 10.1038/ng.3695 27723756 PMC5626933

[B53] Watts S. C. Ritchie S. C. Inouye M. Holt K. E. (2019). FastSpar: Rapid and scalable correlation estimation for compositional data. Bioinformatics 35, 1064–1066. 10.1093/bioinformatics/bty734 30169561 PMC6419895

[B54] Yilmaz B. Juillerat P. Øyås O. Ramon C. Bravo F. D. Franc Y. (2019). Microbial network disturbances in relapsing refractory Crohn’s disease. Nat. Med. 25, 323–336. 10.1038/s41591-018-0308-z 30664783

[B55] Zhou X. Stephens M. (2012). Genome-wide efficient mixed-model analysis for association studies. Nat. Genet. 44, 821–824. 10.1038/ng.2310 22706312 PMC3386377

