# Supplementary material for: Network-based quantitative trait linkage analysis of microbiome composition in inflammatory bowel disease families
Source: Front Genet. 2023 Jan 23;14:1048312. doi: 10.3389/fgene.2023.1048312 (PMC9901208; doi:10.3389/fgene.2023.1048312)
Supplement: Supplementary file 2 [file Image1.pdf]

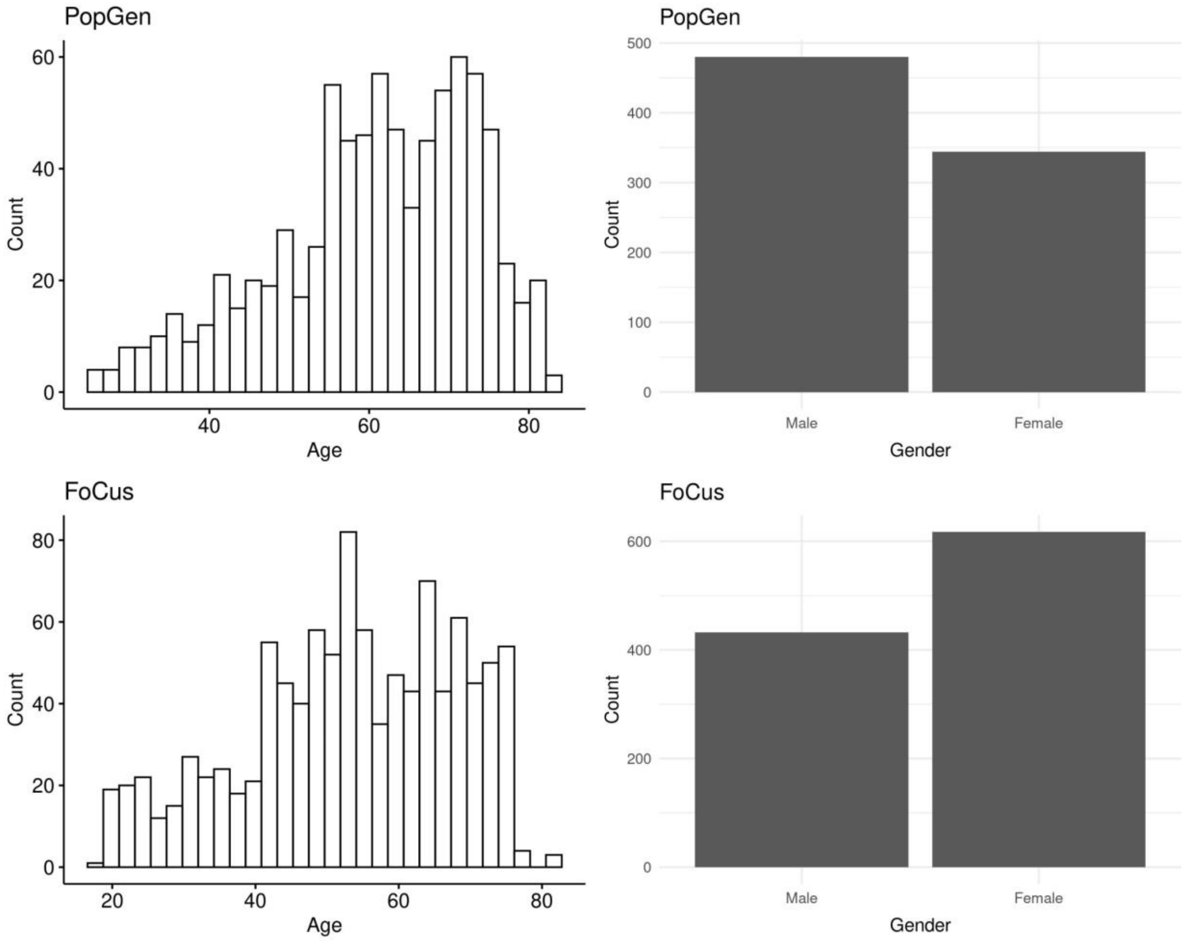

Supplementary Figure 1. Distribution of age and gender in the PopGen and FoCus control cohorts.

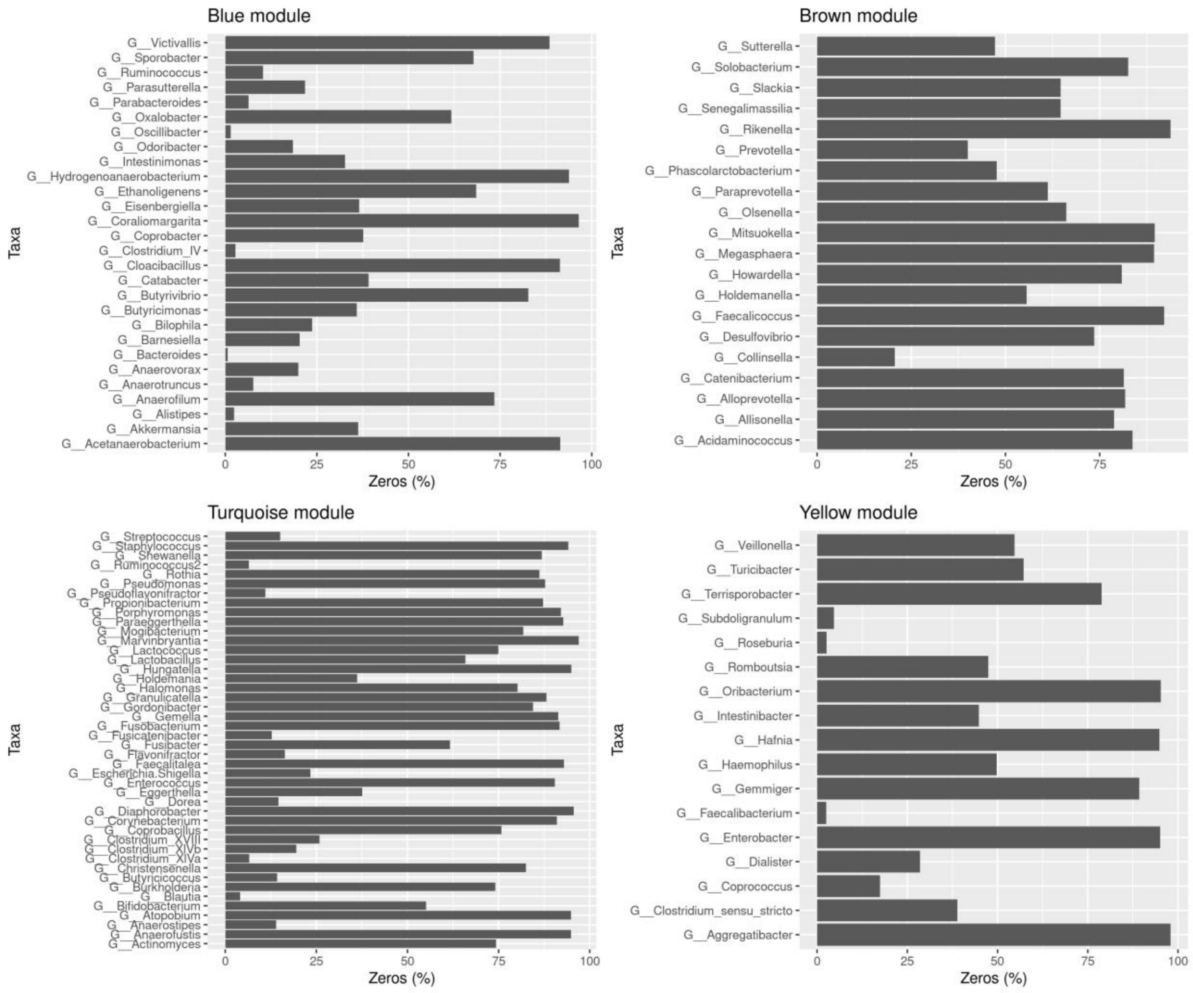

Supplementary Figure 2. Percentage of individuals in the combined control cohort with an abundance of zero for each of the genera constituting microbial co-occurrence network modules.

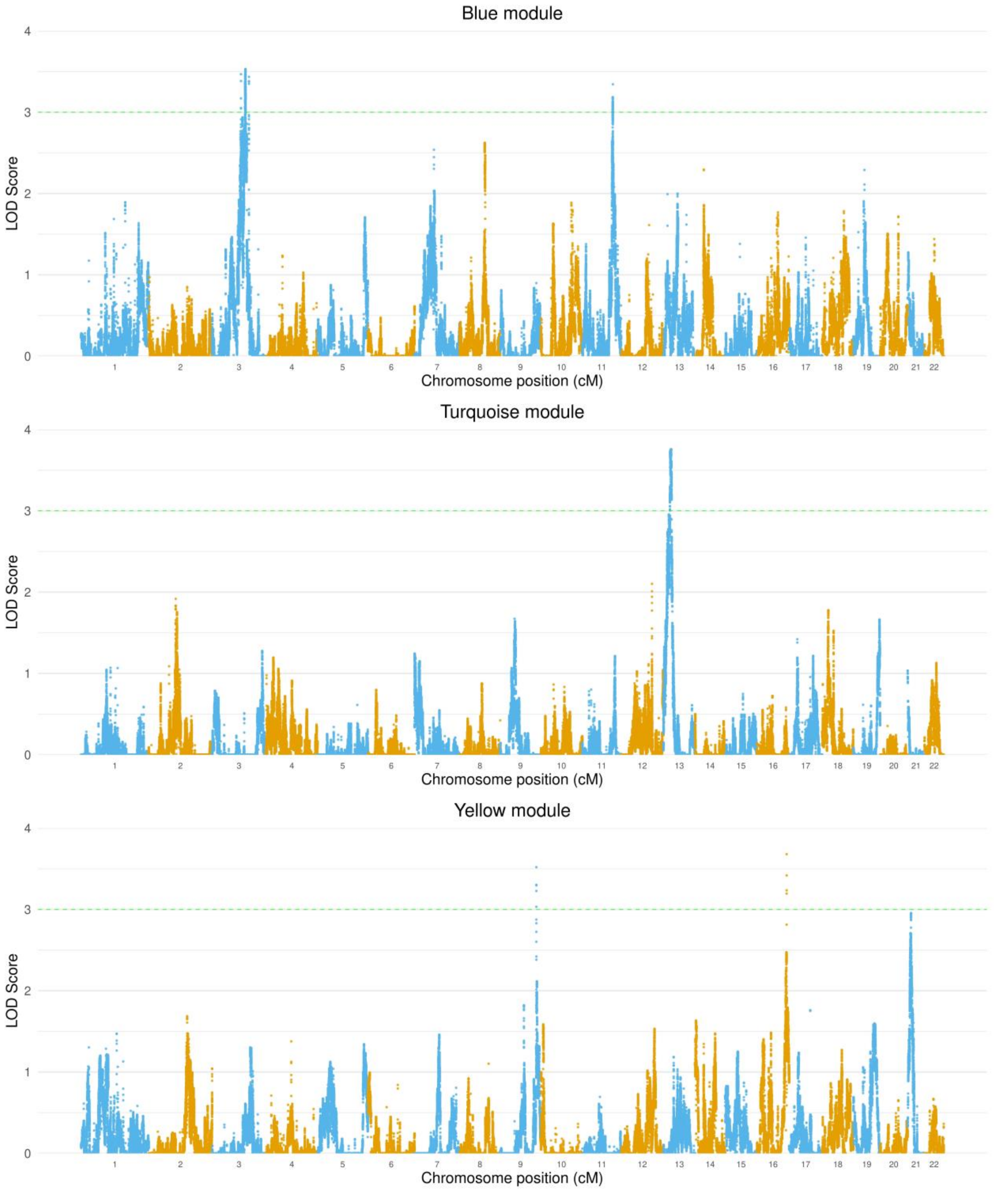

Supplementary Figure 3. Genome-wide linkage analysis of module-specific QTs derived from microbial co-occurrence network modules. The threshold for genome-wide significance (LOD >3) is marked by a green line.
